# Rare Variant of Carcinoma Prostate Masquerading as Benign Prostatic Hyperplasia

**DOI:** 10.7759/cureus.4504

**Published:** 2019-04-20

**Authors:** Sachin Khanduri, Tariq Imam, Mazhar Khan, Shahla Khan, Ahmad Umar Khan

**Affiliations:** 1 Radiology, Era's Lucknow Medical College and Hospital, Lucknow, IND; 2 Radiodiagnosis, Era's Lucknow Medical College and Hospital, Lucknow, IND

**Keywords:** prostatic cancer, ductal adenocarcinoma

## Abstract

Prostatic ductal adenocarcinoma, is an uncommon entity in the spectrum of prostatic carcinoma. Clinically it is different from common prostatic acinar adenocarcinoma. It is usually more aggressive than prostatic acinar adenocarcinoma. We are presenting a case report on prostatic ductal adenocarcinoma, a cystic variant of prostatic carcinoma in a 55-year-old man who complained of obstructive urinary symptoms with mildly raised prostate-specific antigen (PSA). On further evaluation in our radiology department a cystic lesion with enhancing polypoidal soft tissue component was noted in prostatic parenchyma. Histopathology confirmed the diagnosis of ductal adenocarcinoma.

## Introduction

As hair becomes white in men, the risk of prostatic malignancy increases. Prostatic cancer is the second most common cause of death in men usually having an indolent course. World Health Organization (WHO) in 2016 classified epithelial tumor of the prostate in various subcategories like acinar and ductal adenocarcinoma and almost all prostatic malignancies develop from glands as acinar adenocarcinoma. Pure ductal adenocarcinoma, formerly known as “endometriod adenocarcinoma”, is a clinical and histological variant that arises from ducts and represents only 0.2%-0.4% of all prostatic malignancies. The incidence of ductal adenocarcinoma has been increasing over each decade at approximately the same rate as acinar carcinoma [1]. Various studies reported aggressive nature of ductal adenocarcinoma than acinar prostatic carcinoma, including higher stage and metastasis and higher mortality rate at the time of presentation [2-3].

Cystic prostatic carcinoma is a rare entity with few cases reported in the literature [4]. Malicow and Pachter were first to describe a case of prostatic ductal adenocarcinoma in 1967 and it was suggested that tumor’s origin was of prostatic utricle (uterus masculinus) having “endometrial features” [5]. Prostatic ductal adenocarcinoma is thought to arise from primary ducts of the prostate with various studies over the last few decades using microscopic, immuno-histochemical and ultra structural methods supporting this article.

In our case report, we represented a 55-year-old man who came to our hospital with abdomen-pelvic complaints such as pain in abdomen and obstruction in urine with few episodes of hematuria and he was further clinically investigated and radiopathologically diagnosed as prostatic ductal adenocarcinoma: a cystic variant of prostatic carcinoma.

## Case presentation

A 55-year-old man came to our hospital with complaints of vague abdominal pain, burning micturition, hematuria, increased frequency and urgency of urine, more during night and pain during defecation since four months. He was referred to the urology department where the clinical examination was done including digital rectal examination which revealed an enlarged prostate. Further, more hematological investigations were done with no abnormal findings aside from prostate-specific antigen (PSA) which was mildly raised with a value of 12.4 ng/ml. He had no remarkable medical history.

The patient was further investigated in our radiology department where an abdominal ultrasonography and intravenous pyelography (IVP) was done. On ultrasonography, he was diagnosed with moderate hydroureteronephrosis with chronic cystitis with significant post void urine and increased volume of the prostate which measured approximately (75)cc with grade-III prostatomegaly and a large cystic swelling within prostatic parenchyma. On IVP it was diagnosed as moderate hydroureteronephrosis with significant residual post void urine.

**Figure 1:**
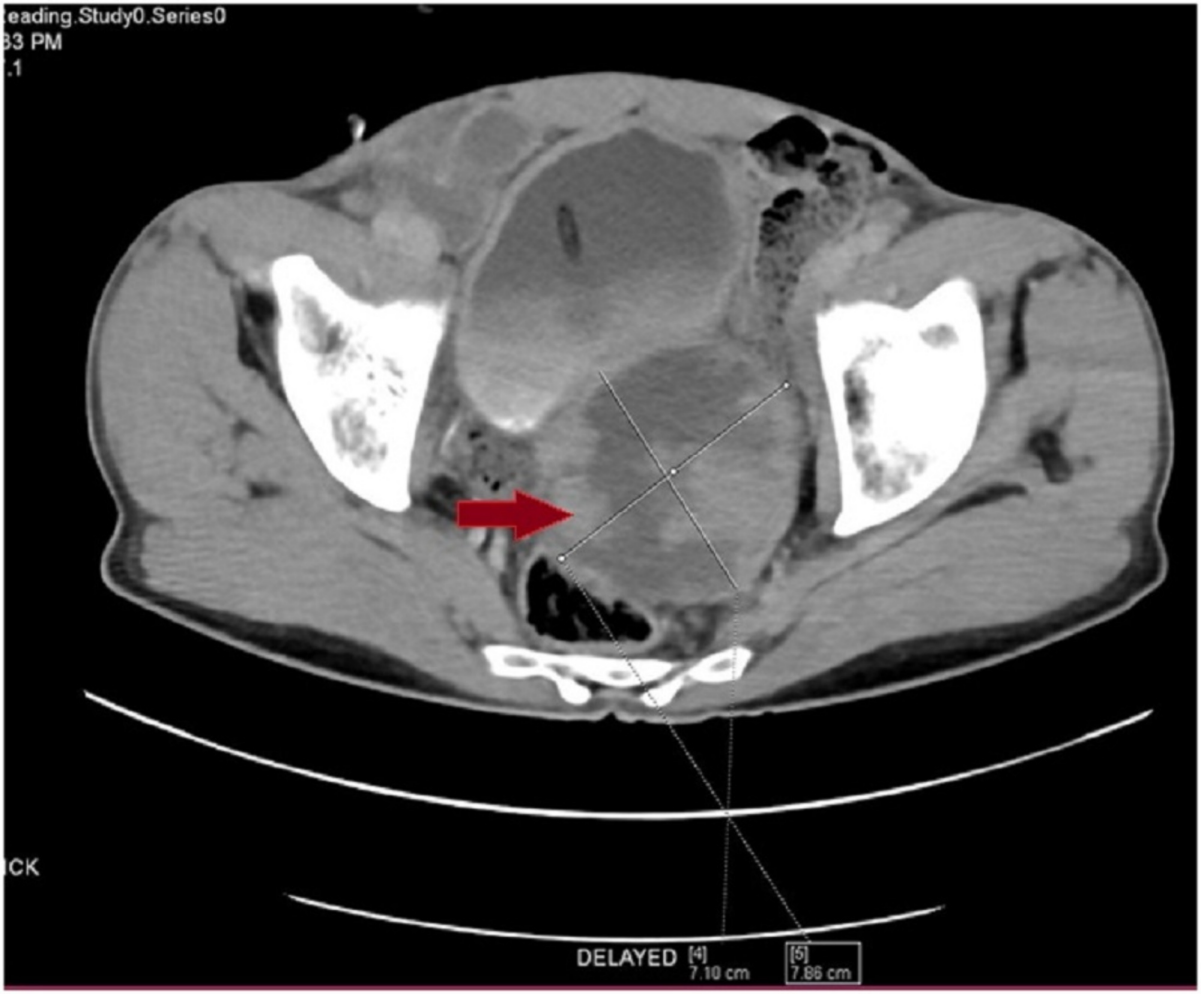
Contrast-enhanced computed tomography with axial image of delayed phase showing large intra-prostatic cyst (red arrow)

Contrast-enhanced computed tomography (CECT) was done with axial images of delayed phase which showed well defined large cystic lesion measuring approximately 7.8 x 7.1 cm in the mid and left pelvis likely arising from the prostate. Lesion demonstrated multiple enhancing polypoid soft tissue components along the wall. The lesion abutted urinary bladder anteriorly, rectum medially and levator ani muscle posteroinferiorly with loss of fat planes as shown in Figure 1.

**Figure 2:**
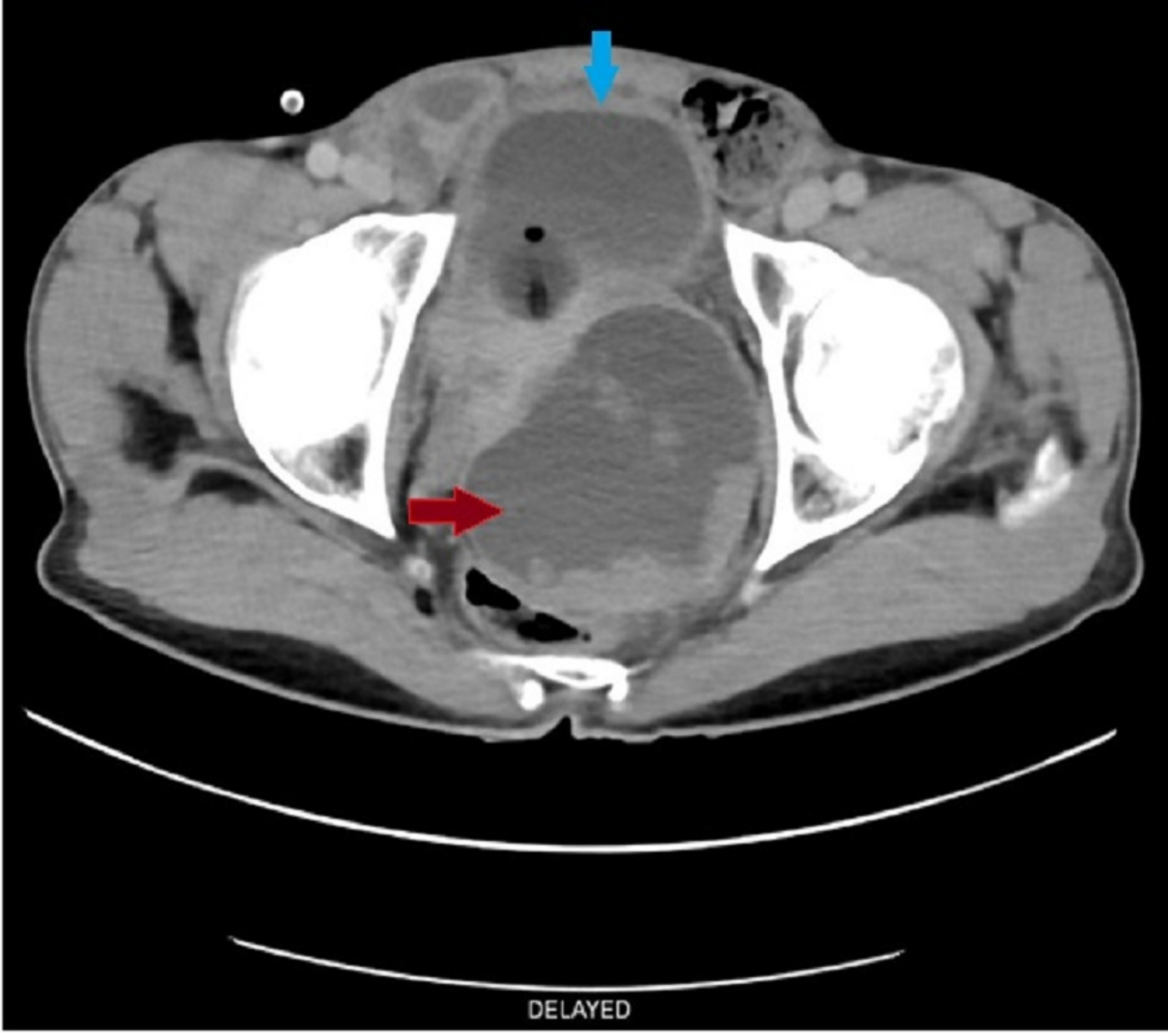
Contrast-enhanced computed tomography with axial image delayed phase showing mid circumferential wall thickening of the urinary bladder with Foley’s bulb (blue arrow) and prostatic cyst (red arrow)

There was also mid circumferential wall thickening of the urinary bladder with few internal iliac lymph nodes as shown in Figure 2.

Bone scan and computed tomography (CT) of chest, abdomen, and pelvis were negative for metastasis.

**Figure 3:**
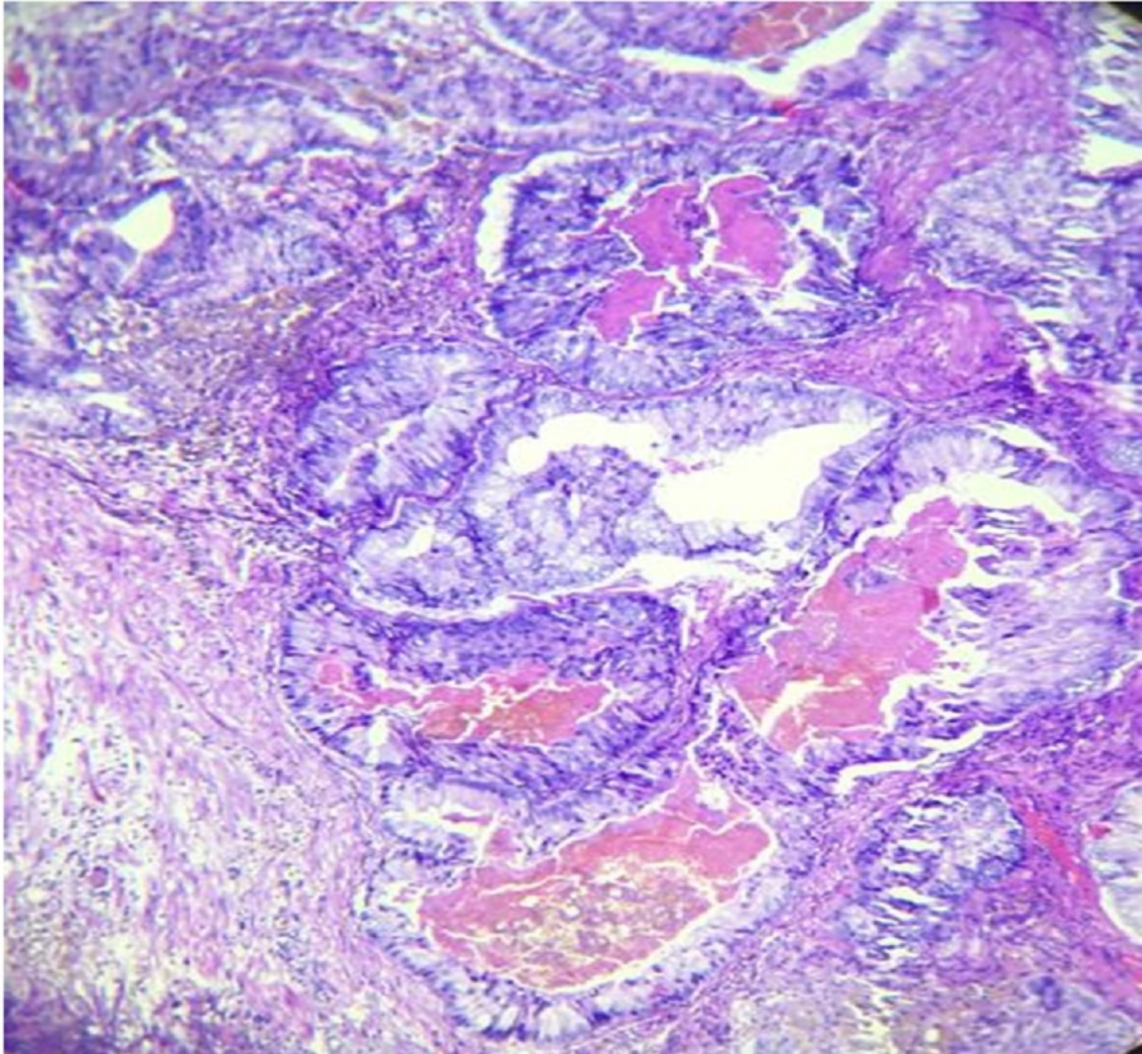
Section shows tumor with glands as weak as papillary fronds (H&E 100x) H&E: Hematoxylin and Eosin stain

**Figure 4:**
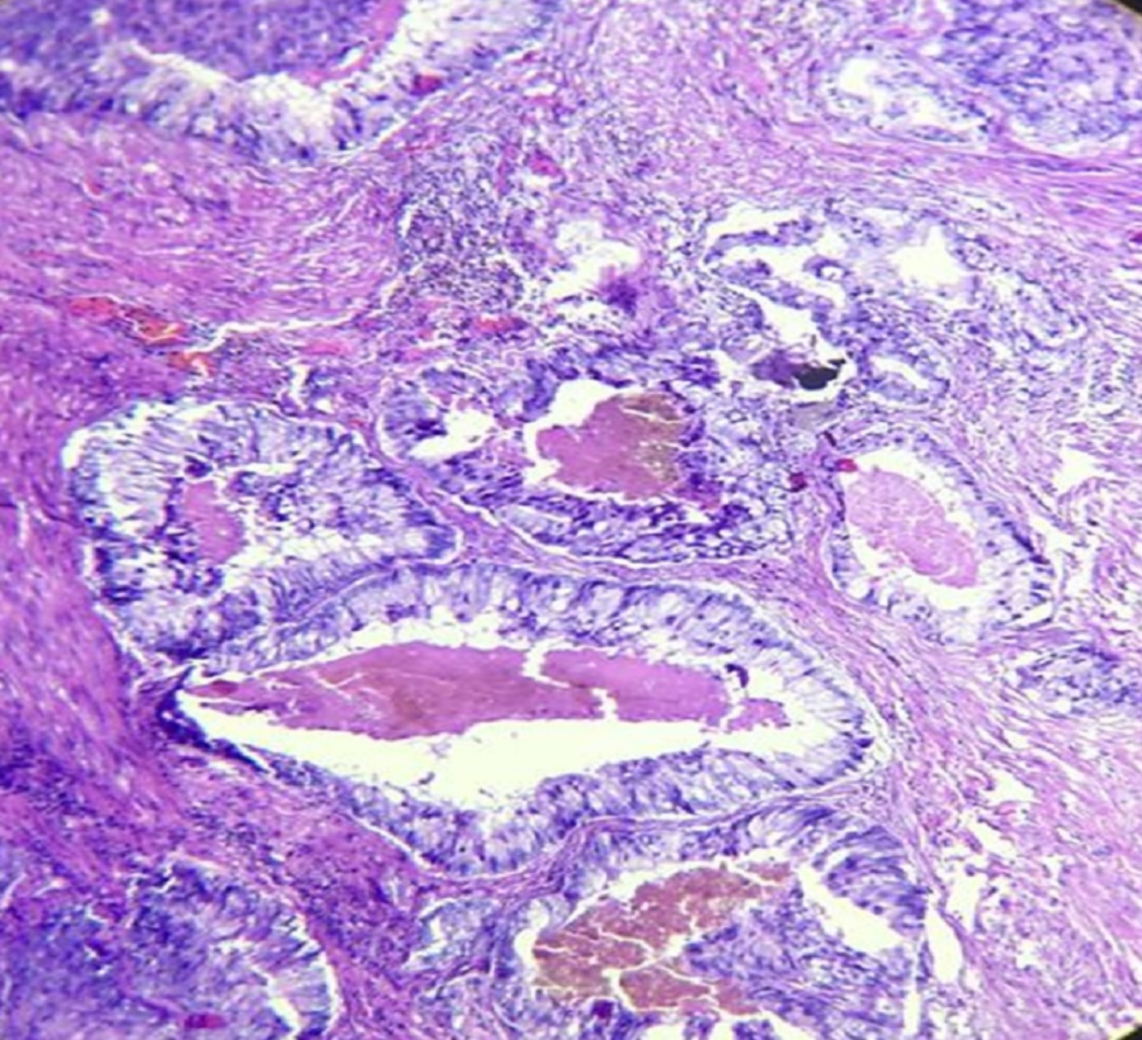
Specimen shows tumor glands with scanty intervening stroma, intraluminal eosinophilic secretion, and amphophilic cytoplasm with anisonucleosis (H&E 400x) H&E: Hematoxylin and Eosin stain

Histological sections from the specimen, as seen in Figures 3-4, consist of 19 out of 22 prostatic transurethral resection of prostate (TURP) chips which showed tumor composed of medium-sized glands with irregular outline and a smooth inner surface and scanty intervening stroma. Ducts were lined by pseudostratified columnar epithelium with abundant amphophilic cytoplasm and intraluminal eosinophilic secretion. Anisonucleosis was seen. On histopathological sections, diagnosis of ductal adenocarcinoma of the prostate was made with Gleason score 5+4=9, grade group V (WHO 2016 classification).

The patient underwent combination treatment open radical retropubic prostatectomy along with androgen blockade drugs, bicalutamide, and goserelin for six months. An 8-10 cm incision was made (well below the belt-line) in the skin between the umbilicus and the top of the pubic bone. The pelvis was then explored and the important structures such as the urinary bladder, prostate, urethra, blood vessels, and nerves were identified.

The prostate along with cyst was removed from the urethra below and the bladder above, and the bladder and urethra were reconnected. The yellowish color fluid was found in the cyst. The blood vessels leading to and from the prostate were divided and tied off. A catheter running through the penis into the bladder was typically required for at least a week after surgery. A surgical drain was left in the pelvis to allow drainage of blood and other fluid. Additional components of the operation include: - Lymphadenectomy: Left sided internal iliac lymph nodes were removed. Prostate cancer often spreads to nearby lymph nodes in the early stages, especially the sentinel lymph node.

- Nerve-sparing surgery was attempted to protect the cavernous nerves of the penis, which control erection. These nerves are very thin and fragile and run next to the prostate and may be destroyed during surgery, leading to impotence.

- Bone scan and CT chest, abdomen, and pelvis were negative for metastasis; radiotherapy was not given to the patient. Serum PSA level was within normal range of 4 ng/ml and under follow up.

## Discussion

Carcinoma of the prostate is the most common form of cancer in men and is the second most common cause of death [6]. Ductal adenocarcinoma of the prostate was first reported as endometrial carcinoma of prostatic urticle in 1967 [5]. Ductal adenocarcinoma of the prostate with or without a mixed acinar component represent a rare entity among prostatic carcinoma variants.

Tumor represents pathologically by tall columnar, pseudostratified epithelium with papillary architecture and macroscopically, it appears to be exophytic with villous growth and can involve the surrounding pelvic structure. Men with prostatic ductal adenocarcinoma are likely to present with locally advanced disease, although lymph nodes appear to be comparable between ductal and acinar pathology [1].

It is suggested that in prostatic ductal adenocarcinoma presenting with normal or slightly raised serum PSA level except in metastatic disease due to growth in pre-existing ducts or urethra which provide a route of egress of cellular secretions [3]. Thus, there are higher chances of under diagnosis of prostatic ductal adenocarcinoma due to normal to low level of serum PSA level.

Histological differential diagnosis includes prostatic intraepithelial neoplasia and intraductal carcinoma (well circumscribed, malignant lumen spanning lesion of prostatic epithelial origin, with intact basal cell layer and found in close proximity to invasive cancer) [7].

Prostatic ductal adenocarcinoma tends to metastasize to some unusual locations like dural metastasis to falx and visceral which may be due to the propensity of prostatic ductal adenocarcinoma to disseminate via hematogenous route rather than lymphatics [8].

## Conclusions

Purely cystic prostatic ductal adenocarcinoma is a rare entity. In our case report, we diagnosed our patient with prostatic ductal adenocarcinoma on radiological basis and was further proved by histopathological examination. Our patient was diagnosed having ductal adenocarcinoma of the prostate with Gleason score 5+4=9, grade group V (WHO 2016 Classification). Our patient underwent a combination of surgical resection and androgen deprivation therapy and is under follow-up. More efforts are being made from our behalf to contribute knowledge regarding tumor biology, to understand normal to slightly raised serum PSA levels, and to assess its response to therapy.
